# Recent Progress of Spectroscopic Probes for Peroxynitrite and Their Potential Medical Diagnostic Applications

**DOI:** 10.3390/ijms241612821

**Published:** 2023-08-15

**Authors:** Zixin Liu, Shanyan Mo, Zhenming Hao, Liming Hu

**Affiliations:** Beijing Key Laboratory of Environmental and Viral Oncology, Faculty of Environment and Life, Beijing University of Technology, Beijing 100124, Chinamo@bjut.edu.cn (S.M.); haozhenming@emails.bjut.edu.cn (Z.H.)

**Keywords:** peroxynitrite, fluorescent probes, intracellular imaging, in vivo imaging, medical diagnostic

## Abstract

Peroxynitrite (ONOO^−^) is a crucial reactive oxygen species that plays a vital role in cellular signal transduction and homeostatic regulation. Determining and visualizing peroxynitrite accurately in biological systems is important for understanding its roles in physiological and pathological activity. Among the various detection methods, fluorescent probe-based spectroscopic detection offers real-time and minimally invasive detection, high sensitivity and selectivity, and easy structural and property modification. This review categorizes fluorescent probes by their fluorophore structures, highlighting their chemical structures, recognition mechanisms, and response behaviors in detail. We hope that this review could help trigger novel ideas for potential medical diagnostic applications of peroxynitrite-related molecular diseases.

## 1. Introduction

**Scheme 1:**
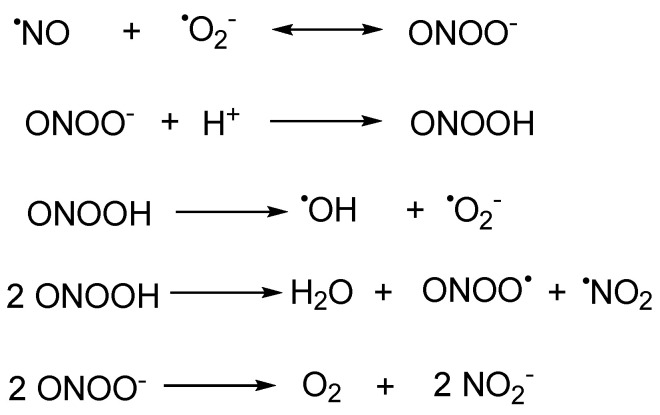
The biogenesis of peroxynitrite and its transformations with other major reactive species.

Peroxynitrite (ONOO^−^) is a kind of reactive oxygen species (ROS) generated by the rapid reaction of nitric oxide (NO) and a superoxide anion free radical (O_2_·^−^) in the absence of enzyme catalysis, which has strong oxidation, nucleophilic, and nitration properties [1]. It occupies crucial roles in the transformations of other major reactive species. (Scheme 1) Its pKa value is 6.8 [2], and the half-life is approximately 1 s [3,4] at pH 7.4. ONOO^−^ can react with a variety of bioactive substances (such as protein, nucleic acid, lipid, etc.) with very high reactivity. In addition to its oxidation, nucleophilic, and nitration properties, ONOO^−^ can also be converted into higher activity secondary free radicals, including hydroxyl radicals (·OH), nitro radicals (·NO_2_), and carbonate radicals (CO_3_·^−^), which further react with biomolecules and ultimately lead to cell death.

Based on these properties, peroxynitrite exhibits two effects with different directions. In the living system, when the ONOO^−^ remains at a level which is under normal physiological conditions, it serves as an indispensable physiological activator and signaling molecule. However, when the concentration of ONOO^−^ elevates, the excess ONOO^−^ will turn the redox state of the cell to a pro-oxidant state [5,6]. Eventually, serious inflammation and disease will be induced, for example, rheumatism, hepatic disease, neurodegenerative disease, cancer, and so on [7,8,9,10]. Therefore, it would be of great significance to develop a method which could accurately detect ONOO^−^ and explore the physiological role of ONOO^−^ in living systems.

In comparison to other ONOO^−^ detection methods (positron emission computed tomography (PET), computed tomography (CT), magnetic resonance imaging (MRI), and genetically encoded indicators) [11], spectroscopic detections, especially fluorescent probes, possess advantages such as excellent temporal and spatial resolution, simple operation, high sensitivity and selectivity, and non-destructive and in situ real-time visualization of biological samples [12,13,14,15].

So far, a variety of reviews on ONOO^−^ fluorescent probes have been published [12,13,14,15]. This review focuses on the fluorophore structure in the ONOO^−^ fluorescent probe molecules with their potential medical diagnostic applications. Herein, we categorized, analyzed, and discussed the recently reported organic probes according to their fluorophore core, including xanthene (rhodamine, rhodol, and fluorescein), cyanine (and hemicyanine), coumarin, malononitrile-based [Dicyanomethylene-4H-pyrans (DCM), dicyanoisophorone (DCI or DCO) dyes, quinoline-malononitrile (QM)], 2-benzothiazoleacetonitrile-based dyes, and naphthalimide. In particular, we summarized the key factors of the ONOO^−^-responsive probes, such as chemical structures, responsive pathways, emission wavelength, dynamic range of fluorescence response, response time, ONOO^−^ detection range, detection limit, ONOO^−^ production pathways in the biosystem, and bioimaging objects. We believe that researchers will benefit from this review when they rationally design ONOO^−^ fluorescent probes, thus contributing more excellent theranostic studies in relating areas. We will review other spectroscopic probes for the detection of ONOO^−^.

## 2. Fluorescent Probes

### 2.1. Xanthene as Fluorophore Core

Xanthene dyes can be categorized as fluorescein, rhodol, and rhodamine based on the type of the substituents on the 3- and 6-position [16]. They are well known because of their switchable fluorescent off–on flexibility. Xanthene dyes can produce fluorescence wavelengths above 510 nm, reaching far-red areas depending on the conjugative substituents. Thus, they are of widespread use in optical diagnostic research [17].

The triggers of ONOO^−^-responsive probes with xanthene as a fluorophore core were generally built on (1) oxidation of the hydrazide (**Xan1–Xan15**) [18,19,20,21,22,23,24,25,26,27,28,29,30,31,32], (2) oxidative cleavage of the substituents at the hydroxyl or amino group (**Xan 16–Xan 28**) [33,34,35,36,37,38,39,40,41,42,43,44,45], (3) oxidation of pyrylium (**Xan 29–Xan 33**) [46,47,48,49,50], and (4) oxidation of the hydrogenated xanthene (**Xan 34–Xan 37**) [51,52,53,54] and others (**Xan 38–Xan 41**) [55,56,57,58]. The key elements of the ONOO^−^ response of probes are summarized in Table 1.

#### 2.1.1. Hydrazide Oxidative Xanthene Probes

In 2002, Guo et al. reported a spiro form hydrazide rhodamine (**Xan 1**) [18] as the ONOO^−^ fluorescent probe. The hydrazide probe was colorless and non-fluorescent. Upon treating with ONOO^−^, the spiro hydrazide group was oxidized, releasing a highly fluorescent rhodamine B. The response finished in as fast as 30 s. Meanwhile, the detection limit was only 24 nM. The response avoids interference from the 10^−5^ M Cu(II) ion. Thus, it represents the rapid, sensitive, and specific fluorescent detection of ONOO^−^.

**Figure 1:**
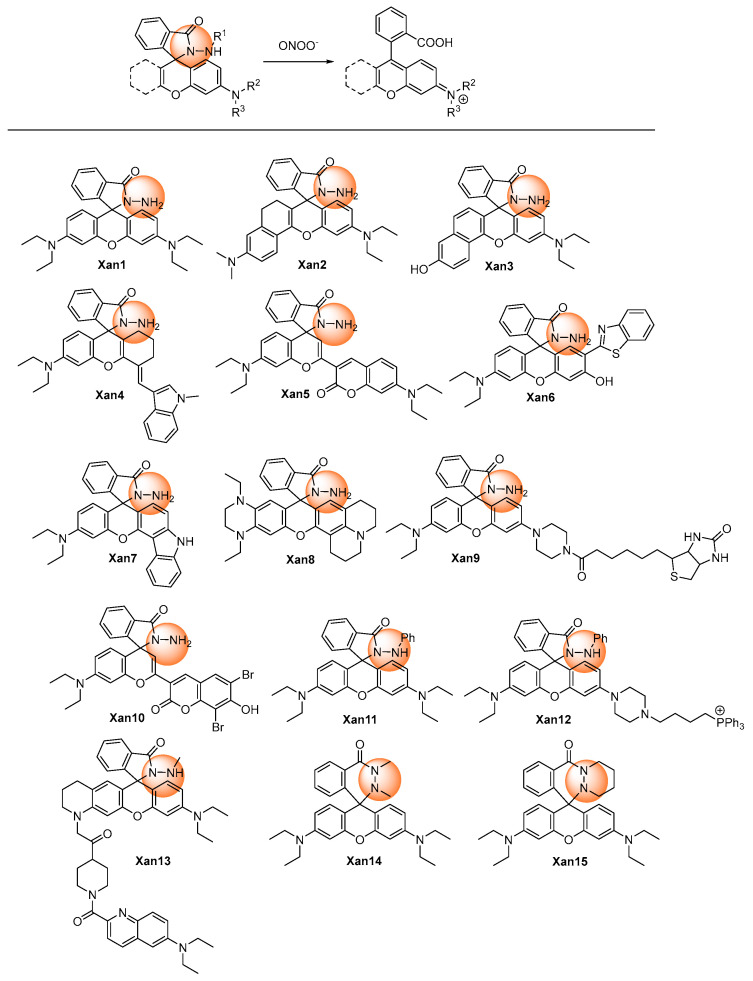
Chemical structures of the hydrazide oxidative xanthene probes.

Based on the recognized pattern and the easy structurally modification character of rhodamine, a series of related probes were developed, aiming to improve the performance of different aspects of the response (Figure 1). Longer emissive wavelengths (up to the NIR range) were obtained with more conjugate groups installed in **Xan 2–5** [19,20,21,22], **Xan 8** [25], and **Xan 10** [27]. Dual-channel fluorescence was afforded when coumarins were introduced to the rhodamine ring (515/700 nm for **Xan 5** and 631/669 nm for **Xan 10**), making the response produce more information. Ratiometric fluorescence was realized in **Xan 6** [23] and **Xan 7** [24] with the introduction of a 2-(2′-hydroxyphenyl)benzothiazole group and a 4-hydroxycarbazole group, respectively, in which the intensity of the original band disappeared with the generation of a new band with a longer wavelength. Large Stokes shift and excellent lysosome-targeting ability were achieved with the engineering of a fused tetrahydroquinoxaline ring, making **Xan 9** [26] capable of detecting both peroxynitrite and lysosomal pH. Sodium-dependent multivitamin transporter (SMVT)-targeted ability was acquired by introducing the biotin group for Xan 9, making it possible to detect the peroxynitrite in head and neck cancer cells.

If a phenyl group was introduced into the hydrazide (**Xan 11–Xan 12**) [28,29], the response time would prolong to 10 or more minutes, presumably due to the steric-hindrance-caused decreased reactivity. It should be noted that, with alkyl substituent groups in both nitrogens of the hydrazide, the cyclic hexahydropyridazin probes (**Xan 14–Xan 15**) [31,32] displayed a faster response rate than the alkyl-substituted (**Xan 13**) [30] or phenyl substituted hydrazide xanthene (**Xan 11–Xan 12**). The response was usually specific, without interferences from a lot of metal ions and other reactive oxygen and nitrogen species. [28,29,30,31,32]

Peroxynitrite generated from different cells, such as HeLa, RAW264.7, HepG2, HSC-2, and Cal-27, could be detected by hydrazide xanthenes. Meanwhile, these probes could detect peroxynitrite in zebrafish and mouse models. These outstanding performances made hydrazide xanthenes capable of revealing the important roles of peroxynitrite in many kinds of diseases, such as respiratory infectious diseases and inflammation in the future.

#### 2.1.2. Oxidative Cleavage of the Recognition Groups to Release Xanthene Probes

**Figure 2:**
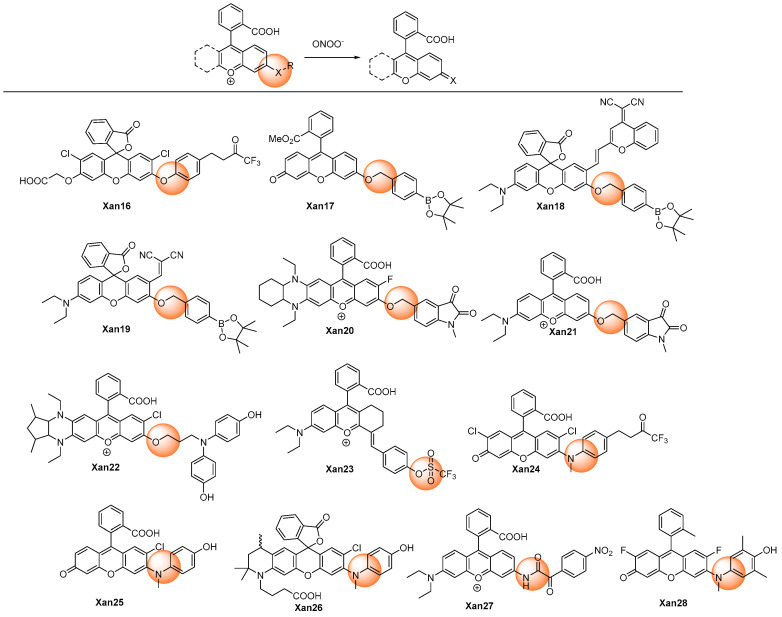
Chemical structures of the oxidative cleavage xanthene probes.

Utilizing the oxidative ability of peroxynitrite, the recognition groups at the 2- or 6-hydroxyl or amino group of xanthenes derivative could be cleaved to release xanthenes with high fluorescence (Figure 2).

Yang et al. developed the HKGreen series of rhodamine probes (**Xan 16** [33] and **Xan 24** [41]) for detecting peroxynitrite with the employment of the trifluoromethyl ketone as the recognition group, which involved dioxirane rearrangement and oxidative O- or N-diarylation. The response was highly selective and sensitive, with high-fold fluorescent enhancement, though the response time was relatively long (>15 min).

While using the benzyl boronates moiety as a recognition group, **Xan 17–19** [33,34,35] exhibited quite different responsive behaviors upon reactive species. **Xan 17** [33] reacted not only to peroxynitrite but also with hypochlorite and hydrogen peroxide, though with different second-order rate constants. However, **Xan 18** [34] and **Xan 19** [35] responded exclusively to peroxynitrite, even when hypochlorite and hydrogen peroxide were at much higher concentrations. Nevertheless, all of them displayed obvious fluorescent enhancement and low detection limits, and thus they were all further employed for fluorescent imaging in biosystems involving diseases such as drug-introduced liver injury.

The 1-methylindoline-2,3-dione group can also be employed as the recognition moiety for the specific detection of peroxynitrite (**Xan 20** [36] and **Xan 21** [37]). The mechanism involving intramolecular cyclization of peroxynitrite with indoline-2,3-dione, rearrangement, and 1,6-elimination was proposed [36]. Leveraging the probes, the two-photon (TP) in vivo NIR imaging technique was applied to observe the peroxynitrite level in a mouse tumor, a tumor onset on the second day, a kidney injury of zebrafish, and the microvessels of mouse brains with strokes [37].

Xanthenes with the electron-donating groups substituted phenyl groups as recognition groups (**Xan 16** [33], **Xan 24** [41], **Xan 25** [42], **Xan 26** [43], and **Xan 28** [45]) produced very high fluorescent enhancement, probably due to their better quenching effect.

#### 2.1.3. Oxidation of Pyrylium

Yuan et al. discovered an aminophenyl-substituted pyrylium as a highly sensitive and selective scaffold towards peroxynitrite after the screening of nineteen dyes and then further modified it to a FRET probe (**Xan 29**) [46] with TP absorption. After the response, the pyrylium emission band at 651 nm disappeared, and a coumarin characteristic emission band at 473 nm was enhanced. Detailed response mechanisms involving nucleophilic addition, oxidation, elimination, and hydrolysis reactions on chromenylium fluorophore were proposed and verified by MS spectra. Although the destroyed-type response led to the decrease of the emission wavelength, the combination technique of the ratiometric measure and TP imaging made it possible to specifically and rapidly visualize the peroxynitrite in an inflamed mouse model. Furthermore, the detection limit was as low as 11.3 nM, which was at a super level among the peroxynitrite probes. Subsequently, similar structures were synthesized for different applications. Gong et al. reported esterified **Xan 30** [47] with better membrane penetrability and mitochondria targeting ability, which could image the peroxynitrite in the acute liver injury model in living cells. Li et al. introduced a piperazine ring to respond to the pH and finally realized the fluorescent imaging of the cellular peroxynitrite level as well as the mitophagy behavior [48].

**Figure 3:**
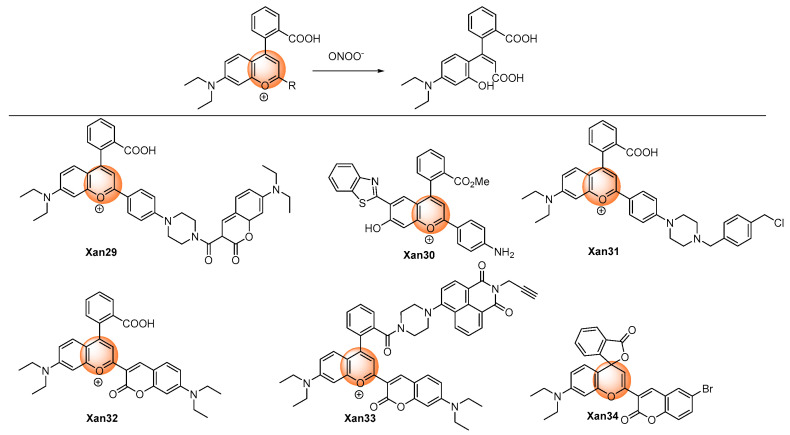
Chemical structures of the pyrylium oxidative xanthene probes.

Yuan et al. performed an original structure–activity relationship study of the substituents at the recognition site. (Figure 3) They discovered that pyrylium involving aryl substituents with strong electron-withdrawing groups could improve the sensitivity; meanwhile, pyrylium involving aryl substituents with strong electron-donating groups could improve the selectivity. Hence, they designed a coumarin, which was a not strong electron-withdrawing and -donating group, substituted pyrylium (**Xan 32**) [49] to satisfy the high requirements of both selectivity and sensitivity, and the results showed that an outstanding detection sensitivity of 4.1 nM of the detection limit as well as a high 130-fold ratiometric emission signal were realized. Employing the probe, the changing content of peroxynitrite in the diseases model involving nonalcoholic fatty liver and drug-induced liver injury was successfully visualized to unfold the functionality of a related enzyme. Zhou et al. introduced a naphthimide fluorophore in the xanthene carboxylic position. After the response, both coumarin and naphthimide fluorescence were produced to output a multicolor signal. The probe **Xan 33** was applied for the early detection and evaluation of arthritis [50].

However, a similar structure–activity relationship study conducted by Tang et al. produced totally different results and response mechanisms, in which electron-withdrawing groups were installed in the 6-position of coumarin moiety. They found that because of the installation of the electron-withdrawing groups in the 6-position of coumarin, **Xan 34** produced 4-(2-carboxylphenyl)-7-diethylaminocoumarin (λem = 520 nm) and 3-hydroxy-6-bromocoumarin (non-fluorescent) as products after the response [51]; nevertheless, **Xan 32** and **Xan 33** produced 3-carboxyl-7-diethylaminocoumarin (λem = ~468 nm) and a ring-opening product of pyrylium (non-fluorescent). Furthermore, **Xan 34** could also detect biothiols by the additional recognition site on coumarin.

#### 2.1.4. Oxidation of Hydrogenated Xanthene

**Figure 4:**
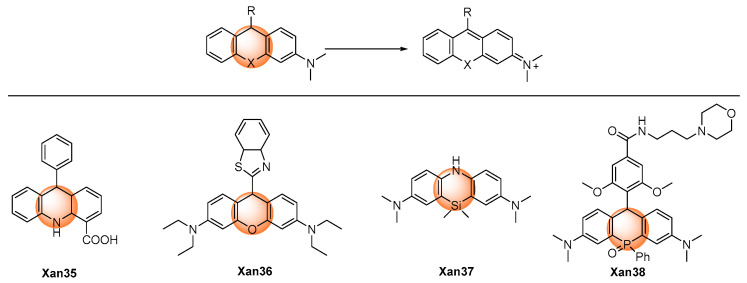
Chemical structures of the oxidative hydrogenated xanthene probes.

Peroxynitrite can oxidize t non-fluorescent hydrogenated xanthene to produce highly fluorescent aromatic products. (Figure 4) Gong et al. developed 9,10-dihydroacridine **Xan 35** as the peroxynitrite detection probe. An over 100-fold fluorescence enhancement could be achieved after reacting with peroxynitrite. The probe was utilized to detect intracellular peroxynitrite [52]. Similar O-, Si-, and P- hydrogenated rhodamine systems were also reported. **Xan 36–37** [53,54] displayed a very fast response speed (<20 s); for **Xan 38** [55], the relatively low response speed was probably due to the low reactivity caused by the presence of the electron-withdrawing phosphonic group. Nevertheless, **Xan 35–38** [52,53,54,55] all exhibited very low detection limits at the nanomolar level, and they were all applied to fluorescent imaging of cell endogenous peroxynitrite.

#### 2.1.5. Others

**Figure 5:**
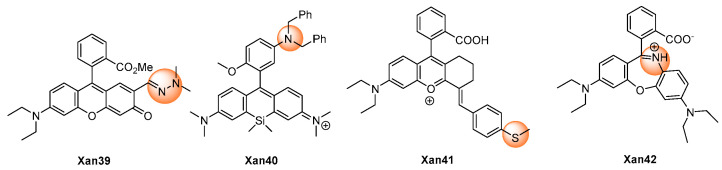
Chemical structures of the other xanthene probes.

Wu et al. described a Rhodol-based probe, **Xan 39** [56], which introduced 1,1-dimethylhydrazone as a peroxynitrite recognition group. (Figure 5) The probe was non-fluorescent as a result of the rotational vibration of the C=N bond. Using the oxidative ability of peroxynitrite, the hydrazine was oxidatively cleaved into the corresponding aldehyde with significant fluorescence. The response exhibited a low detection limit (57 nM) with a short response time (<60 s). The probe was applied in the fluorescent imaging of exogenous and endogenous peroxynitrite in living cells.

Miao et al. reported **Xan 40** [57] as a peroxynitrite off–on probe. The probe showed little fluorescence because of the photo-induced electron transfer (PeT) quenching effect of the 3-dibenzylaminophenyl group. Upon reaction with peroxynitrite, a benzyl group was removed and formed an N-oxide product, and the fluorescent was turned on.

Li et al. released the study on **Xan 41** [58] as a peroxynitrite probe, in which the fluorescence was turned off by the intramolecular charge transfer (ICT) effect of the 4-methylthiophenyl group. After the response with peroxynitrite, the thiol ether was transformed into sulfoxide and discontinued the ICT effect, thus recovering the fluorescence and realizing the detection of the peroxynitrite concentration.

Zhang et al. reported a novel rhodamine probe **Xan 42** with the dibenzo[1,4]oxazepine core as the responsive moiety [59]. Synthesized by the reaction of rhodamine with hydroxylamine, the probe was of little fluorescence at 672 nm. However, after the treatment with peroxynitrite, oxazines was generated with high fluorescence. The probe was used to monitor the peroxynitrite level in living cells.

### 2.2. Dicyano-Based Compounds as Fluorophore Core

Dicyano-based compounds are characteristic of their donor–π–acceptor structure, which endows them with large Stokes shifts and excellent photostability as a result of the ICT process. In addition, this sort of chromophore was generally easily synthesized and structurally modified. Thus, great attention has been attracted towards dicyano-based compounds to build probes with different functionalities [60].

**Figure 6:**
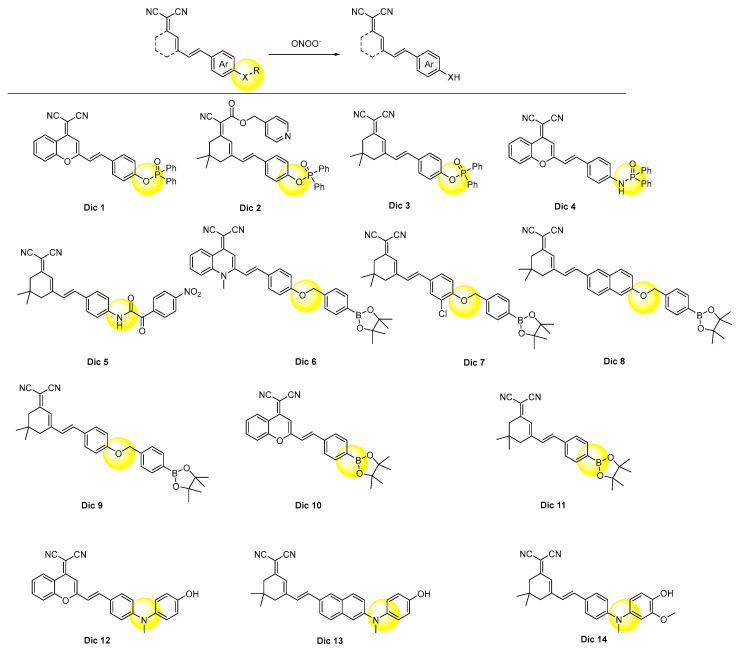
Chemical structures of the dicyano-based probes.

The designing rule for dicyano-based peroxynitrite probes was a consensus, which was described in Figure 6. In general, the responsive groups, such as diphenylphosphonyl, benzyl boronates, and 4-hydroxyphenyl, were modified on the donor moiety to stop the ICT process. The response with peroxynitrite would break the links between the donor moiety and the response groups and release the dicyano-based chromophores with strong fluorescence.

As summarized in Table 1, **Dic 1–4** [61,62,63,64], with diphenylphosphonyl as a recognition group, took more than 10 min to respond, which was relatively longer than those of **Dic 5–14** [65,66,67,68,69,70,71,72,73,74]. This was probably due to their high intrinsic structural stability. However, their detection selectivity and sensitivity were not reduced. Thus, they were employed for fluorescent imaging of the exogenous peroxynitrite in living cells. Among them, **Dic 4** were further used to manifest the changing peroxynitrite concentration in the rat epilepsy model with the aid of two-photon fluorescent technology [64].

**Dic 5** [65] with the 4-nitrophenyl oxoacetyl group as the responsive unit showed a much faster response rate (<2 s) than **Dic 1–4**, but its detection limit was at a similar level (81 nM). The probe was used for fluorescence imaging of the endogenous peroxynitrite in zebrafish and mice.

All of the benzyl boronates derived dicyano-based probes **Dic 6–10** showed analogous response times to each other. Interestingly, **Dic 6** [66] and **Dic 10** [70] only displayed green-channel fluorescence, although they both have an extra conjugate phenyl ring compared **Dic 7** [67] and **Dic 9** [69], respectively. The boronate group of **Dic 10–11** was oxidized to the hydroxyl group in situ by peroxynitrite, and the transformation generated the donor, thus forming the ICT process and producing fluorescence.

The response rates of the probes **Dic 12–14** [72,73,74], which employed 4-hydroxylphenyl as a masking group, were all found to be ultrafast, which were 5, 25, and 1 s, respectively. This phenomenon was in accordance with those of **Xan 25–26** [42,43] and **Xan 28** [45], suggesting the great advantage of this mask group. Possessing the superior detecting sensitivity and selectivity, the probes **Dic 12–14** were applied to visualize the peroxynitrite in different diseases, including inflammation, acute liver injury, and Parkinson’s disease [72,73,74].

### 2.3. Coumarin as Fluorophore Core

The research history of coumarin (also known as 1-benzopyran-2-one or 2H-chromen-2-one) was more than 200 years. Plenty of extensive investigations have been performed to modify the weak fluorescent parent coumarin to its derivatives with different desired photophysical properties, with a considerable amount of them now very active in the commercial market [75].

**Figure 7:**
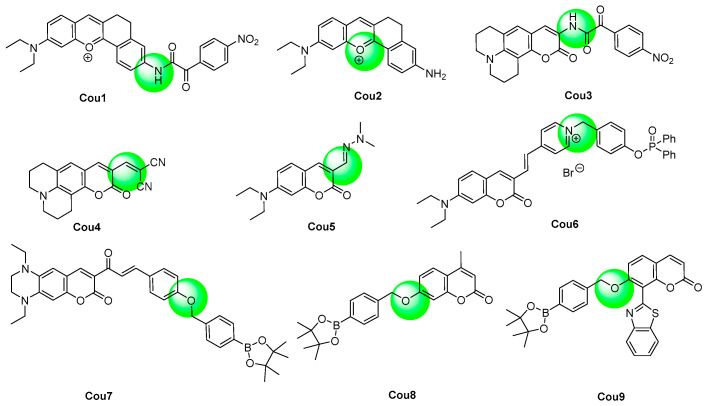
Chemical structures of the coumarin probes.

Inserting the electron-donors in the 7-position leads to a bathochromic shift to the emission wavelength; in addition, a donor–π–donor structure was formed, which facilitates the use of itself to design the ICT type probes by further introducing an electron-acceptor recognition group. (Figure 7) Xie et al. adopted this strategy and synthesized **Cou 1** [76]. The 4-nitrophenyl oxoacetyl recognition group reacted with peroxynitrite rapidly and produced the deprotected product **Cou 2** [77]. They used **Cou 1**, together with the two-photon fluorescent imaging technology, to visualize the peroxynitrite produced in the mitochondria in an anthracycline-induced cardiotoxicity mouse model. However, Li et al. reported that the deprotected product, **Cou 2**, also further reacted with peroxynitrite in 5 s in the concentration range of 0.064–0.64 μM, and the resulting nitration products were confirmed by ESI-MS analysis. The 3-position of coumarin could also be introduced with electron-donors to generate the donor–π–donor structure. Wei et al. developed **Cou 3** as the peroxynitrite probe using the 4-nitrophenyl oxoacetyl group as a recognition moiety [78]. The fluorescence of **Cou 3** was quenched but could be quickly recovered with eight-fold enhancement after the response with peroxynitrite. The probe was used to image exogenous peroxynitrite formation in living cells in a biosystem.

The electron effect of the substituents of the 3-position of 7-dialkylaminocoumarin derivatives decided their emission properties. The existence of an electron-acceptor can cause a strong ICT effect and fluorescence, and the stronger the electron-withdrawing ability the group owned, the longer the emission wavelength and stronger fluorescence the probes owned. If the electron-withdrawing ability changed, the fluorescent property would change accordingly. For example, the formyl group is a medium-ability electron-withdrawing group. If it was transformed into stronger electron-withdrawing groups, the emission wavelength of the product, **Cou 4**, would increase [79]. In reverse, after the response with peroxynitrite, the C=C bond of **Cou 4** broke and generated the aldehyde product. The response was completed in a very short time with high selectivity and sensitivity to peroxynitrite. If the aldehyde group was reacted with hydrazine, the hydrazone product **Cou 5** would emit only little fluorescence. However, after the reaction with peroxynitrite, the fluorescence would recover [80].

The ICT process is very strong in the quaternized pyridinium probe **Cou 6** [81]. After the addition of peroxynitrite, the diphenyl phosphinate was eliminated, and the product owned a very weak ICT process. The fluorescence undergoes a hypochromatic shift from 643 nm to 538 nm, and the emission ration displays a 153-fold increase. The probe was applied to detect the peroxynitrite in living cells.

Parthiban et al. reported a coumarin–chalcone hybrid peroxynitrite probe **Cou 7** containing a tetrahydroquinoxaline ring [82]. The probe displayed a large Stokes shift of 149 nm. The aryl boronate group was employed as the recognition group for peroxynitrite. The probe exhibited exceptional speed and sensitivity in detecting peroxynitrite. Palanisamy described another coumarin probe **Cou 8** with a 7-position aryl boronate group as the response moiety, and the probe was applied to fluorescence imaging of peroxynitrite in a high-fat diet-induced obese mouse model [83].

Wang et al. reported a coumarin probe **Cou 9**-based 7-position benzyl borate as a recognition group. The probe exhibited weak ICT and weak fluorescence at 421 nm [84]. After the reaction with peroxnitrie, a strong ICT and FRET process was turned on and led to an incredible 1200-fold enhancement of the fluorescence. The probe was used for fluorescent imaging of the content of peroxnitrie in cancer cells.

### 2.4. N-Substituted Coumarin as Fluorophore Core

**Figure 8:**
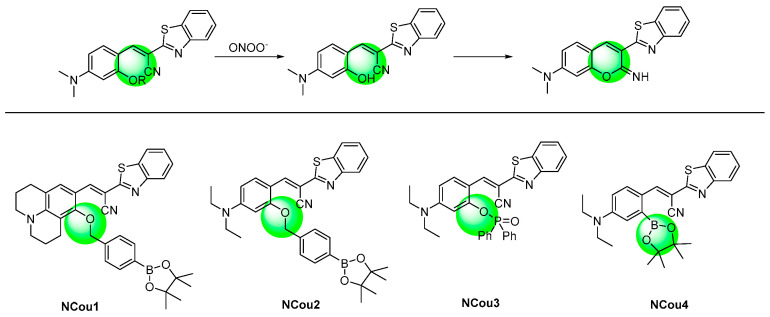
Chemical structures of the N-substituted coumarin probes and their responsive mechanism.

As analogues for coumarin dyes, 2-(benzo[d]thiazol-2-yl)phenylacrylonitrile derivatives exhibited longer emission wavelengths than the related coumarins. (Figure 8) In particular, 2-(benzo[d]thiazol-2-yl)-3-(2-hydroxyphenyl)acrylonitrile derivatives (**NCou 1–3**) [85,86,87] served as the precursors for iminocoumarin, and they exhibited aggregation-induced emission luminogens (AIEgens) in aqueous conditions. Upon the response with peroxynitrite, 2-(benzo[d]thiazol-2-yl)-3-(2-hydroxyphenyl)acrylonitrile would be generated, which would further transform into iminocoumarin in situ. The probes were applied for the fluorescent imaging of cell exogenous and endogenous peroxynitrite, though the response rate was usually relatively slow.

The hydroxyl group was also converted from the borate group. The probe **NCou 4** exhibited high speed and sensitivity in detecting peroxynitrite [88]. The detection limit of the probe for peroxynitrite was 0.83 nM.

### 2.5. 1,8-Naphthalimide as Fluorophore Core

**Figure 9:**
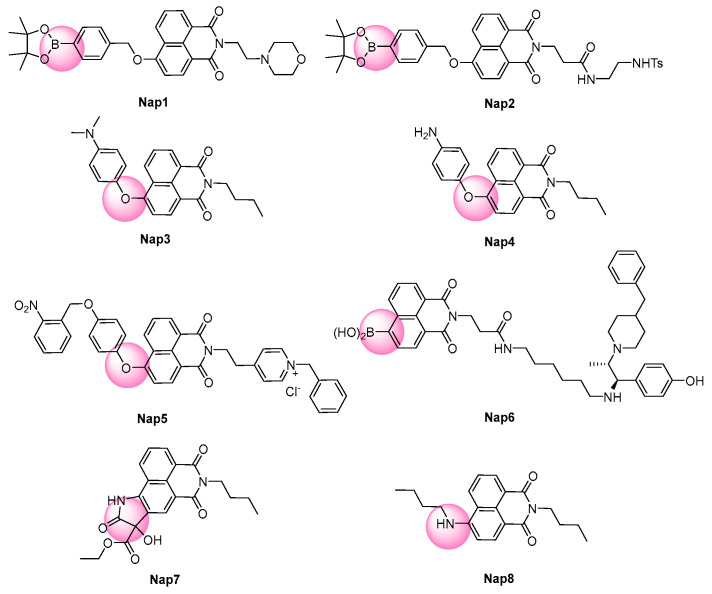
Chemical structures of the 1,8-naphthalimide probes.

1,8-Naphthalimide and its derivatives have been employed in a variety of analyte-detecting applications owing to their good chemical stability and outstanding photophysical properties [89]. (Figure 9) The switch to control the off-and-on state of the fluorescence was usually installed on the 4- or 5-position of the hydroxy or amino group at the 1,8-naphthalimide. Through protection with a recognition group on the hydroxy or amino group, the ICT process stopped. After the reaction with peroxynitrite, the recognition group was removed, the ICT process was restored, and the fluorescence was enhanced.

Wang et al. reported a 4-hydroxyl-1,8-naphthalimide derivative probe **Nap 1** targeting lysosomes with benzyl borate as the response group for the detection of peroxynitrite. After the addition of peroxynitrite, the fluorescence at 550 nm was greatly increased [90]. The response finished in a very short period (<70 s), without interference by a lot of common metal ions and ROS, and the detection limit was only 130 nM. The probe was used for the visualization of the changing levels of peroxynitrite in three types of acute liver injury mouse models. Sun et al. described a similar probe, **Nap 2**, with a p-toluenesulfonamide group used as the endoplasmic reticulum (ER)-targeted group [91]. With the aid of ratiometric two-photon fluorescent technology, they revealed the increased exogenous peroxynitrite level at ER in the hippocampus of the depressive mouse.

The aminophenol group could also be used to prevent the ICT process of the 5-hydroxy-1,8-naphthalimide and quench its fluorescence. Meanwhile, it was easily oxidized and de-arylated. Thus, it was very suitable to be employed as the recognition group for the peroxynitrite probe. **Nap 3** [92] and **Nap 4** [93] probes were built on the above strategy. Both of them exhibited good sensitivity and specificity over peroxynitrite, and they were used for the fluorescent imaging of the exogenous and endogenous peroxynitrite of the living cells and zebrafish or *C. elegans*.

To enclose the ROS level during the ferroptosis process in the mitochondria, Xie et al. built a photocontrol peroxynitrite probe **Nap 5** [94]. The fluorescence could be turned on only when the probe was simultaneously exposed to peroxynitrite and light irradiation. This avoided the false fluorescent signal generated outside the mitochondria. The ability to target mitochondria was endowed by the lipophilic cation group. Based on the solid evidence, the authors revealed the changing peroxynitrite level and its possible biological source during ferroptosis and suggested that the mitochondrial peroxynitrite was closely related to ferroptotic progression.

The N-methyl-D-aspartate (NMDA) receptor acted as a significant role in memory-related molecular biology. Lee et al. developed a 1,8-naphthalimide-based probe, **Nap 6**, to visualize peroxynitrite near the NMDA receptor in neuronal cells and hippocampal tissues [95]. The oxidation of the boric acid by peroxynitrite led to the generation of a hydroxy group at the 5-position of 1,8-naphthalimide. The fluorescence was increased after the response. The cytotoxicity of **Nap 6** was negligible, and its sensitivity and selectivity to peroxynitrite upon other ROS and RNS were extremely high. Thus, it could be used to investigate the cellular functions related to peroxynitrite near NMDA receptors.

Xie et al. described an oxindole derivative probe **Nap 7** for the detection of peroxynitrite [96]. The probe could specifically and quickly respond to peroxynitrite. In addition, it was able to cross the blood–brain barrier. Therefore, it was used to visualize the peroxynitrite level in live animals to disclose the cerebral peroxynitrite stress state in the 4-month-old Alzheimer’s disease (AD) mouse model.

Zeng et al. discovered that peroxynitrite could oxidize 4-akylamino-1,8-naphthalimide **Nap 8** and cause a reduction in fluorescence [97]. The ratiometric behavior could be used to detect the concentration of peroxynitrite. The recognition was highly selective and sensitive and can be used to sense the peroxynitrite in living cells and zebrafish.

### 2.6. Cyanines as Fluorophore Core

Cyanines have of long research history and are widely used in photo diagnostic and therapy applications due to their excellent optical properties as well as their facile structural modification. Meanwhile, cyanines have remarkable biocompatibility; thus, they are often employed in fluorescent imaging-related clinical trials in which Indocyanine Green (ICG) has been approved by the FDA [98,99]. Cyanines are readily accessed by traditional pyridine or cycloalkyl ketone-initiated procedures or by furfurals derivative started protocols which were recently developed by Mo et al. [100].

**Figure 10:**
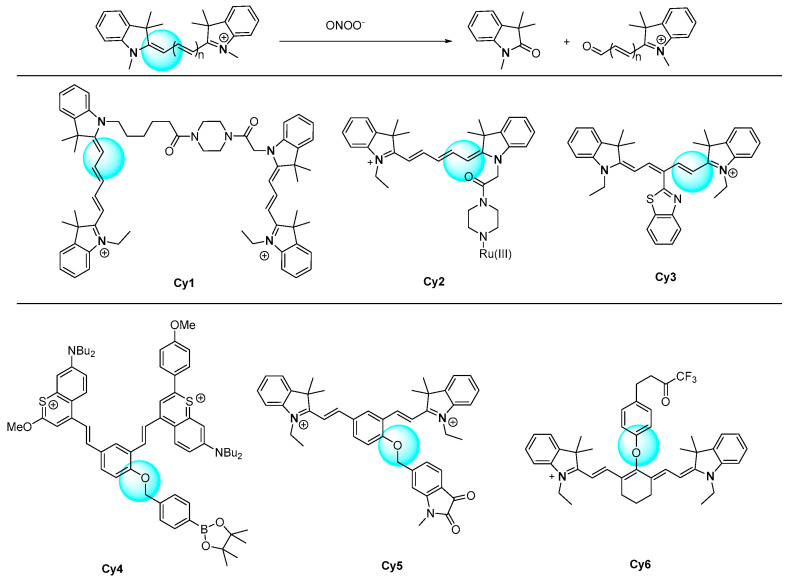
Chemical structures of the cyanine probes.

When exposed to oxidants or nucleophiles, the polymethine bridge of the cyanines could be broken, or form adducts [101]. (Figure 10) Additionally, the longer the bridge is, the more fragile it will be, and the more likely the destructive reactions will happen [102]. Based on this phenomenon, Jia et al. developed Cyanine 3 and Cyanine 5 covalent small-molecule **Cy 1** as the FRET-based ratiometric probe for the detection of peroxynitrite [103]. As the probe response to peroxynitrite, the Cyanine 5 fluorescence band at 660 nm decreased, while the Cyanine 3 band at 560 nm was enhanced. The fluorescent intensity ratio between the two bands realized a 324-fold increase. The detection limit was as low as 0.65 nM, which was an incredible value among those produced from the reported peroxynitrite probes. The probe was used to semiquantitatively detect the peroxynitrite in living cells [103]. In comparison, the probes **Cy 2** [104] and **Cy 3** [105] contained only one cyanine dye. Consequently, their reaction with peroxynitrite resulted in the observation of a relatively smaller wavelength fluorescent signal generated from a cleaved aldehyde fragment.

The conjugated system of phenol-ether center Cyanine 7 was divided in half, which was not capable of emitting typical Cyanine 7 fluorescence. However, when the phenol-ether was fused and turned into the quinone form, the molecule became a heptathine cyanine conjugate system and produced Cyanine 7 fluorescence. Compared to traditional Cy 7anine, quinone Cyanine 7 displayed a generally large Stokes shift of more than 100 nm. In **Cy 4** [106] and **Cy 5** [107], a benzyl boronate and a 1-methylindoline-2,3-dione group were installed in the phenol-ether, which could be fused by peroxynitrite and thus turn the fluorescence on. This fluorescent response was sensitive, exhibiting 55.9 and 25.5 nM of the detection limits, respectively. The probes were applied to visualize peroxynitrite in the mouse model of hepatotoxicity and stroke [106,107].

Huang et al. reported an anisole C4-substituted Cyanine 7 as a peroxynitrite probe **Cy 6** [108]. The probe’s fluorescence was efficiently quenched by the 1,1,1-trifluoro-4-(4-oxyphenyl)butan-2-one group, which produced a clean fluorescent background. After the treatment with peroxynitrite, a dienone product was formed and produced fluorescence at 630 nm. The detection limit was only 9.2 nM. Using the probe, the changing concentration of peroxynitrite in zebrafish and mice under several hypoxic conditions was evaluated, proving that the peroxynitrite produced from hypoxic stress could oxidatively damage cells and tissues.

### 2.7. Half-Cyanines as Fluorophore Core

**Figure 11:**
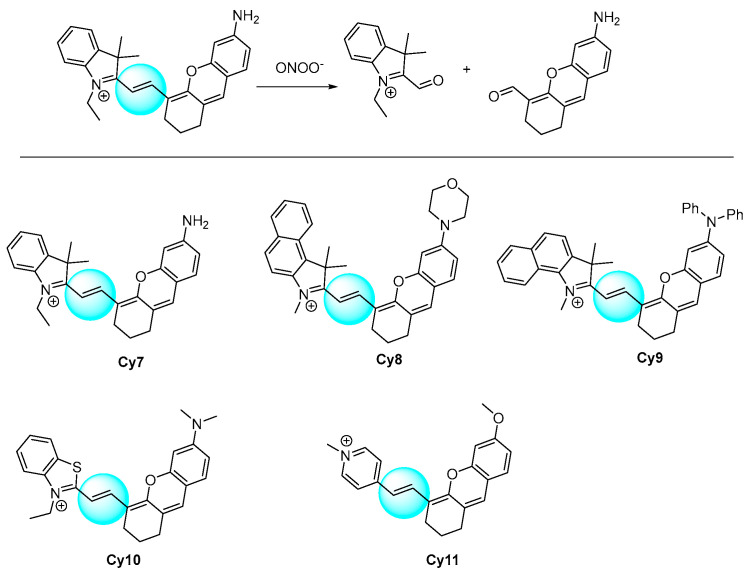
Chemical structures of the half-cyanine probes **Cy 7**–**Cy11**.

As a milestone event, Yuan et al. accidentally obtained a new category of hydroxyl hemicyanine (also known as HDs) by the treatment of chloro-substituted Cyanine 7 with resorcin [109]. The HDs produced NIR range fluorescence, offering an outstanding platform for the establishment of off–on probes. (Figure 11) Although the hydroxyl or amine group is usually used to regulate the optical performance, the C=C bond was a reliable recognition site when the HDs were used to detect peroxynitrite. By the oxidative cleavage of the C=C bond in HDs, an aldehyde product with a lower fluorescent wavelength was generated [110,111,112]. The response was fast and was generally finished in a few minutes. With the **Cy 10** probe, the fluorescence intensity ratio achieved a 1728-fold enhancement after the reaction with peroxynitrite [113]. The aldehyde product was well characterized by MS and NMR, proving the response mechanism. With the use of the probe, the authors realized the ratiometric image visualization of the peroxynitrite in living cells. Very similar spectral behaviors were obtained with similar structure HDs **Cy 11–15** [114,115,116,117,118], regardless of their difference in the quaternized heterocycles and aryl substituents.

**Figure 12:**
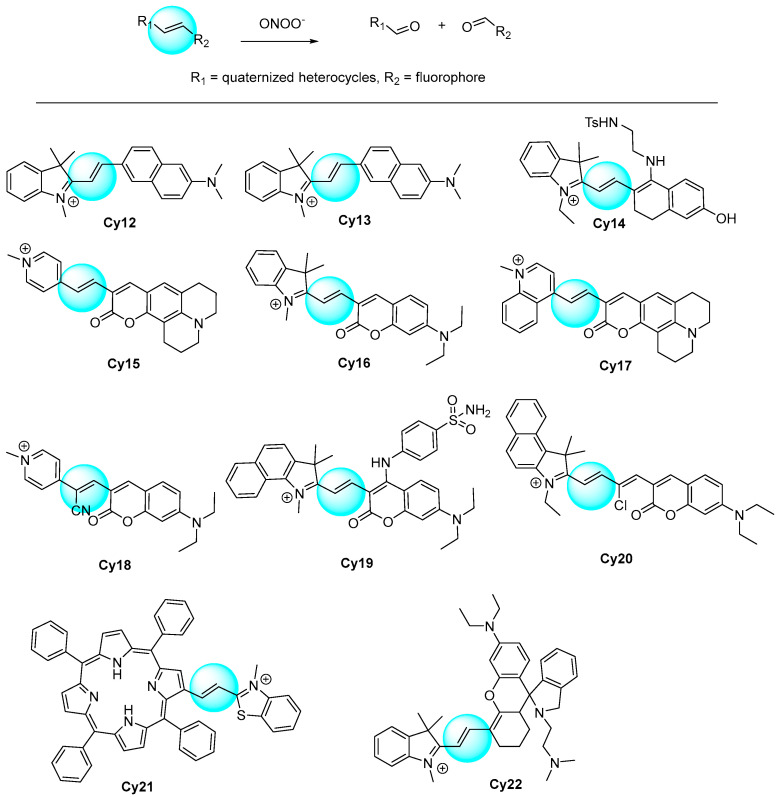
Chemical structures of the half-cyanine probes **Cy 12–Cy22**.

The hemicyanine type of peroxynitrite probes could also be constructed via the condensation of the quaternized heterocycles with various aryl aldehydes. (Figure 12) The aryl group in aryl aldehydes included EDG-substituted naphthalene (**Cy 12** [115] and **Cy 13** [116]) and dihydronaphthalene (**Cy 14** [117]), coumarin (**Cy 15–20** [118,119,120,121,122,123]), porphrin (**Cy 21** [124]), and rhodamine (**Cy 22** [125])). The porphrin and rhodamine groups were relatively less electron-donating than others, leading to longer response times (90 min and 40 min, respectively). However, their detection sensitivity was not reduced (56 and 13 nM, respectively). Depending on the size of the conjugate system, the fluorescence wavelength ranged from 477 to 680 nm. All of these probes were capable of detecting exogenous and endogenous peroxynitrite from living cells.

**Figure 13:**
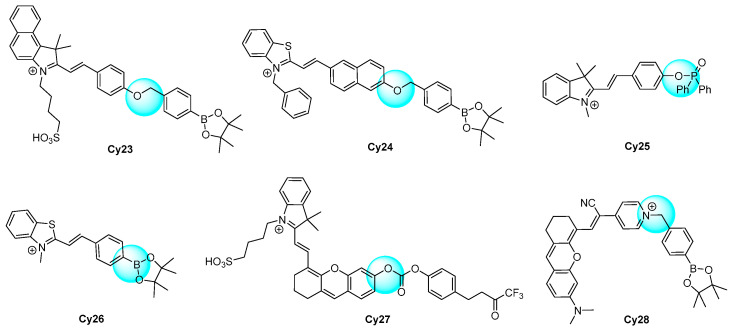
Chemical structures of the half-cyanine probes **Cy 23–Cy28**.

The C=C bonds of the above half-cyanine probes were fused after the reaction with peroxynitrite. (Figure 13) However, when there was another responsive unit in the half-cyanine structure (**Cy 23–29** [126,127,128,129,130,131]), the C=C bond would be maintained, which avoids the hypochromatic shift of the fluorescent wavelength. In these probes, EWG responsive units, including benzyl boronates 1,1,1-trifluoro-4-(4-oxyphenyl)butan-2-one diphenylphosphonyl, were employed to form the ICT process and quench the fluorescence of the probes. The **Cy 23** probe developed by Sonawane et al. displayed good water solubility as a result of the incorporation of a sulfonate group [126]. A remarkable 32-fold fluorescent enhancement was achieved after the response with peroxynitrite. The probe was found to have a mitochondria-targeting ability, and it was applied to investigate peroxynitrite in the zebrafish inflammatory model. The probe **Cy 25** exhibited a wide pH application range of pH 3–9 for the detection of peroxynitrite [128], which was utilized for the fluorescent imaging of peroxynitrite in living cells and thus diagnosing drug-induced liver injury. The probe **Cy 26** could respond to peroxynitrite at a very fast rate with very good selectivity and sensitivity [129]. The authors used the probe to detect the changing concentration of the cell endogenous peroxynitrite and proved that H_2_S was able to scavenge the peroxynitrite produced in living cells. Zhang et al. reported the use of **Cy 27** for the real-time fluorescent and photoacoustic dual-modal imaging of peroxynitrite in the mice tumor, achieving, respectively, 2.1- and 5.3-fold higher signals than the background [130]. Xu et al. developed a dual-responsive probe, **Cy 28**, for the detection of viscosity and peroxynitrite [131]. The fluorescent signals were at 740 nm and 580 nm, respectively. The probe showed low cytotoxicity, very good sensitivity, and high selectivity over a variety of oxidizing species as well as metal, halide, and sulfite ions. The authors employed the probe to realize the fluorescent imaging of peroxynitrite in living HepG2 cells.

**Table 1:** The response behavior of probes.

Reference Number	Dye	λem (nm)	Dynamic Range of Fluorescence Response (Fold)	Response Time	Range (μM)	Detection Limit (nM)	Interference Species (Reactive Species; Anion; Cation; Neutral Species)	ONOO—Production Pathways in the Biosystem	Fluorescent Bioimaging Objects
[18]	**Xan1**	556	NR	<30 s	0.075–3.0	24	H_2_O_2_; NO_2_^−^, NO_3_^−^; Cu^2+^; Cys, Met, GSH, NHOH, Glucose, ascorbic acid, Epinephrine	Aqueous	NR
[19]	**Xan2**	638	80	<180	0–34	45	H_2_O_2_, ClO^−^, ·OH, ·O_2_^−^, ^1^O_2_, tBuOOH, tBuOO·, NO·; NO_2_^−^, NO_3_^−^	Pseudomonas aeruginosa (PAO1)-infected bone marrow-derived neutrophils	HeLa and RAW264.7 cells, mouse
[20]	**Xan3**	630	40	<5 s	0.5–8	17	^1^O_2_, H_2_O_2_, tBuOOH, ·O_2_^−^, ·OH, ^t^BuOO·, OCl^−^; SO_4_^2−^, NO_3_^−^, NO_2_^−^, Cl^−^; K^+^, Na^+^, Mg^2+^, Zn^2+^, Ca^2+^, Al^3+^; Cys, GSH	Cell endogenous	HeLa cells
[21]	**Xan4**	698	100	<2 s	0–100	25	tBuOO·, tBuOOH, NO·, ClO^−^, ·OH, ·O_2_^−^, H_2_O_2_; NO_2_^−^, F^−^, Cl^−^, I^−^, SO_4_^2−^, H_2_PO_4_^−^, SO_3_^2−^, HCO_3_^−^, HS^−^, AcO^−^; Na^+^, Mg^2+^, K^+^, Ca^2+^, Cu^2+^; Cys, Hcy, GSH, Gly, Leu, Lys, Val, Glu	Cell exogenous and endogenous	RAW264.7 cells
[22]	**Xan5**	515/700	NR	~60 s	0–50	59	NO, ClO^−^, ·O_2_^−^, tBuOO·, tBuOOH, ·OH, H_2_O_2_; F^−^, Cl^−^, I^−^, H_2_PO_4_^−^, SO_3_^2−^, HCO_3_^−^, AcO^−^, NO_2_^−^; Na^+^, Mg^2+^, K^+^, Ca^2+^; Gly, Leu, Glu, Val, Lys, Tyr, Cys, Hcy, GSH	Cell exogenous and endogenous	RAW264.7 cells and mouse
[23]	**Xan6**	581	NR	<10	0–18	93	^t^BuOO·, tBuOOH, ·OH, H_2_O_2_, ·O_2_^−^, ^1^O_2_, ClO^−^; NO_3_^−^, NO_2_^−^, Cl^−^, SO_4_^2−^; Zn^2+^, Al^3+^, Na^+^, Mg^2+^, K^+^, Ca^2+^, Fe^2+^, Fe^3+^, Cu^2+^; Cys, Hcy, GSH	Cell exogenous and endogenous	RAW264.7 cells and zebrafish
[24]	**Xan7**	585	NR	<15	0–8.0	10.9	·O_2_^−^, tBuOO·, tBuOOH,·OH, H_2_O_2_, ^1^O_2_; Zn^2+^, Na^+^, Mg^2+^, K^+^, Ca^2+^; Glu, Cys, GSH	Cell exogenous and endogenous	HeLa cells and zebrafish
[25]	**Xan8**	678	100	<2 min	0–70	30	ClO^−^, H_2_O_2_, ·O_2_^−^, tBuOO·,·OH; SO_3_^2−^, HSO_3_^−^, SCN^−^, CO_3_^2−^, S_2_O_3_^2−^, NO_2_^−^, HSO_4_^−^, S_2_O_7_^2−^, AcO^−^, HCO_3_^−^, NO_3_^−^, F^−^, Br^−^, I^−^, Cl^−^, HS^−^; Zn^2+^, Na^+^, K^+^, Ca^2+^, Fe^2+^, Ba^2+^, Cu^2+^; Lys, Val, Asp, Phe, Asn, Ser, Ile, Arg, Tyr, His, Trp, Glu, Ala, Met, Thr, Leu	Cell exogenous and endogenous	RAW264.7 cells
[26]	**Xan9**	575	NR	<1 min	0–10	7	·^1^O_2_, tBuOO·,·OH, tBuOOH, H_2_O_2_, NO, HClO; NO_3_^−^, NO_2_^−^, F^−^, CO_3_^2−^, S^2−^, SO_3_^2−^; Zn^2+^, Na^+^, Mg^2+^, K^+^, Ca^2+^, Fe^2+^, Fe^3+^, Cu^2+^, Mn^2+^; Cys, Hcy, GSH	Cell exogenous and endogenous	HSC-2 and Cal-27 cells, 3D spheroid and mice
[27]	**Xan10**	631/669	10	~1 min	0–20	8	^1^O_2_, H_2_O_2_, ·O_2_^−^, tBuOO·, tBuOOH, ·OH, HClO; Cl^−^, NO_3_^−^, NO_2_^−^, S^2−^; Cys, GSH, HSO_3_^−^	Cell exogenous and endogenous	HeLa and HepG2 cells, zebrafish.
[28]	**Xan11**	580	NR	<10 min	2–20	1.4	H_2_O_2_; SO_4_^2−^, NO_3_^−^, NO_2_^−^; Zn^2+^, Na^+^, Mg^2+^, K^+^, Ca^2+^, Fe^2+^, Fe^3+^, Cu^2+^, Mn^2+^, Hg^2+^; Cys, Met, Thr, Glu, Glucose, Urea, Ascorbic acid	Cell exogenous and endogenous	MCF-7 Cells
[29]	**Xan12**	578	80	<30 min	0–100	55	ClO^−^, NO, H_2_O_2_, ·O_2_^−^, ^1^O_2_, tBuOOH, tBuOO·; NO_2_^−^	Cell exogenous and endogenous	HeLa and RAW264.7 cells
[30]	**Xan13**	574	200	<2 min	0–14	NR	NR	Arginase 1 regulated	RAW264.7 cells and mouse
[31]	**Xan14**	585	NR	<3 s	0–10	0.68	tBuOO·,·OH, ^1^O_2_, ·O_2_^−^, NO, H_2_O_2_, tBuOOH, ClO^−^; Br^−^, SO_3_^2−^, CO_3_^2−^, NO_3_^−^, NO_2_^−^; Zn^2+^, Na^+^, Mg^2+^, K^+^, Ca^2+^, Fe^2+^, Fe^3+^, Cu^2+^, Cu^+^; Cys, Hcy, GSH	Cell exogenous and endogenous	RAW264.7 cells and zebrafish
[32]	**Xan15**	585	NR	<10 s	0–5	61	tBuOO·,·OH, ^1^O_2_, ·O_2_^−^, NO, H_2_O_2_, tBuOOH, ClO^−^; Br^−^, SO_3_^2−^, CO_3_^2−^, NO_3_^−^, NO_2_^−^; Zn^2+^, Na^+^, Mg^2+^, K^+^, Ca^2+^, Fe^2+^, Fe^3+^, Cu^2+^, Cu^+^; Cys, Hcy, GSH,	Cell exogenous and endogenous	RAW264.7 cells and zebrafish
[33]	**Xan16**	521	8	<15 min	0–20	NR	·OH, ^1^O_2_, ·O_2_^−^, NO, ClO^−^, tBuOO·	Cell exogenous and endogenous	Primary cultured neuronal cells
[34]	**Xan17**	518	NR	<40 s	0–20	NR	H_2_O_2_, HClO, tBuOO·, GSH, ·O_2_^−^, ·NO	Doxorubicin introduced	EA.hy926 cells
[35]	**Xan18**	570	NR	<15 min	0–20	52	HClO, H_2_O_2_, ·OH, ^1^O_2_, ·O_2_^−^, tBuOOH, tBuOO·; Cys, Hcy, GSH	APAP-induced liver injury	HepG2 cells, mice
[36]	**Xan19**	573	130	<10 s	0–20	34	HClO, H_2_O_2_, ·OH, ^1^O_2_, ·O_2_^−^, tBuOOH, tBuOO·, NO_2_^−^, NO	Liver ischemia/reperfusion	HL-7702 cells, mice
[37]	**Xan20**	653	NR	<4 min	0–35	72	ClO^−^, H_2_O_2_, ·OH, O_2_^−^, NO; NO_2_^−^; Zn^2+^, Na^+^, Mg^2+^, K^+^, Ca^2+^, Fe^2+^, Cu^2+^	Cancer cell exogenous and endogenous	HeLa and RAW264.7 cells zebrafish, mice
[38]	**Xan21**	557	71	<2 min	0.1–10	NR	ClO^−^, H_2_O_2_, ·OH, O_2_^−^, NO; NO_2_^−^; Zn^2+^, Na^+^, Mg^2+^, K^+^, Ca^2+^, Fe^2+^, Cu^2+^	Brain stroke in mice, LPS induced kidney injury	RAW264.7 cells, zebrafish, mice
[39]	**Xan22**	698	50	<30 min	0–10	3.4	ClO^−^, H_2_O_2_, ·OH, O_2_^−^, NO; NO_2_^−^, S^2−^; Zn^2+^, Na^+^, Mg^2+^, K^+^, Ca^2+^, Fe^2+^, Cu^2+^, Fe^3+^; Hcy, Cys, GSH, vitamin C, Aβ oligomer	Alzheimer’s disease	PC12 cells, mice
[40]	**Xan23**	725	NR	<2 min	0–10	85	ClO^−^, H_2_O_2_, ·OH, ·NO; S^2−^, HS^−^, NO_3_^−^, SO_3_^2−^, SO_4_^2−^, NO_2_^−^; Na^+^, K^+^, Ca^2+^, Fe^2+^, Fe^3+^; Cys, GSH	Myocardium ischemia–reperfusion injury	H9c2 cells, mice
[41]	**Xan24**	535	140	<30 min	0–5	50	tBuOO·, ClO^−^, H_2_O_2_, ·OH, O_2_^−^, ·NO, ClO^−^	Cell exogenous and endogenous	RAW264.7 cells
[42]	**Xan25**	535	290	<5 s	0–4	10	tBuOO·, H_2_O_2_, ·OH, ·O_2_^−^, ·NO	Escherichia coli-challenged	RAW264.7 cells, mouse
[43]	**Xan26**	570	93	<2 s	0–8	-	tBuOO·, H_2_O_2_, ·O_2_^−^, ^1^O_2_,·NO	Acute alcohol-induced liver injury and hepatic ischemic/reperfusion injury	SH-SY5Y cells and live tissues
[44]	**Xan27**	558	14	<30 min	0–16	43	HClO, H_2_O_2_, ·O_2_^−^, HNO, NO, tBuOO·, ·OH; HSO_3_^−^, NO_2_^−^, NO_3_^−^, AcO^−^, SO_4_^2−^; Na^+^, Mg^2+^, K^+^, Fe^2+^, Cu^2+^; H_2_S, H_2_S_2_, Cys, GSH	Drug-induced hepatotoxicity	HepG2 cells,
[45]	**Xan28**	536	1800	<98 s	-	40	ClO^−^, tBuOO·,·OH, ·O_2_^−^, H_2_O_2_, NO	Cellular phagocytosis	RAW264.7 cells
[46]	**Xan29**	651	93	<20 s	0–7.5	11.3	HClO, NO, tBuOO·, ·OH, ·O_2_^−^, H_2_O_2_, tBuOOH, HNO; SO_3_^2−^, NO_2_^−^; H_2_S, H_2_S_2_	Inflamed mouse	HepG2/RAW264.7 cells, hepatic tissue, mouse
[47]	**Xan30**	462	134	<30 min	0–6	1.8	HClO, ·OH, ·O_2_^−^, H_2_O_2_, tBuOOH; SO_3_^2−^, NO_2_^−^; H_2_S, H_2_S_2_, Cys, GSH	Drug-induced acute liver injury	RAW264.7 cells
[48]	**Xan31**	640	NR	<80 s	0.2–1.5	23	^1^O_2_, ClO^−^, ·OH, H_2_O_2_, NO; HS^−^, HSO_3_^−^	Cell endogenous	HeLa and RAW264.7 cells
[49]	**Xan32**	469	130	<25 s	0–7	4.1	tBuOO·, ·OH, tBuOOH, HClO, NO, ·O_2_^−^, H_2_O_2_; NO_2_^−^, SO_3_^2−^; H_2_S, H_2_S_2_, Cys, GSH	Nonalcoholic fatty liver and drug-induced liver diseases	HepG2 and L02 cells, mouse
[50]	**Xan33**	468/526	116	~20 s	0–20	11.6	ClO^−^, ·OH, H_2_O_2_, NO, ·O_2_^−^, HNO; HSO_3_^−^, NO_3_^−^, SO_3_^2−^, NO_2_^−^; H_2_S, Hcy, GSH, Cys	Arthritis	RAW264.7 cells, tissue, mouse
[51]	**Xan34**	520	NR	NR	0–40	1.2	H_2_S, Hcy, Cys, GSH	Acrylamide-induced	PC-12 and HepG2 cells, mice
[52]	**Xan35**	496	100	<5 s	0–2.6	16	^1^O_2_, ClO^−^, H_2_O_2_, ·OH, NO, ·O_2_^−^; NO_3_^−^, NO_2_^−^	Cell endogenous	RAW264.7 cells
[53]	**Xan36**	648	NR	<5 s	0–10	30	^1^O_2_, ClO^−^, H_2_O_2_, ·OH, NO, ·O_2_^−^; Zn^2+^, Na^+^, Mn^2+^, Hg^2+^, Ca^2+^, Fe^2+^, Cu^2+^, Fe^3+^; Cys, GSH, Hcy, NADH	Cell endogenous	HeLa and RAW264.7 cells, mouse
[54]	**Xan37**	760	216	<15 s	0–2	5	^1^O_2_, ClO^−^, H_2_O_2_, ·OH, NO, ·O_2_^−^	Idiopathic pulmonary fibrosis	RAW264.7 cells, mice
[55]	**Xan38**	672	50	<50 min	0–60	80	tBuOO·,·^1^O_2_, ClO^−^, tBuOOH, KO_2_, H_2_O_2_, ·OH; CO_3_^2−^, SO_3_^2−^, SO_4_^2−^, S^2−^, HS^−^, HSO_3_^−^, NO_3_^−^, NO_2_^−^; Zn^2+^, Na^+^, Mg^2+^, Ca^2+^, K^+^, Cu^2+^, Fe^3+^; Hcy, Cys, GSH	Cell endogenous	RAW264.7 cells, mouse
[56]	**Xan39**	571	15	<60 s	0–15	57	NO, tBuOOH, ClO^−^, ·O_2_^−^, ·OH; NO_3_^−^, NO_2_^−^, Cl^−^, S_2_O_3_^2−^, Br^−^, HS^−^; K^+^,Fe^3+^; Cys, Hcy, Cys, GSH, Pro	Cell exogenous and endogenous	HeLa cells
[57]	**Xan40**	680	NR	<30 s	0–4	3	^1^O_2_, H_2_O_2_, ·OH, ·O_2_^−^, ClO^−^, NOC-9; NO_2_^−^; Zn^2+^, Al^3+^, Fe^2+^, Fe^3+^, Cu^2+^, Cu^+^, Na^+^, Mg^2+^, Ca^2+^, K^+^; GSH, DHA	Ischemia–reperfusion injury	RAW264.7, EA.hy926 and INS-1 cells, tissues, mouse
[58]	**Xan41**	548	NR	<5 s	0–26	47	TEMPO, tBuOOH, ·OH, H_2_O_2_, ·O_2_^−^, ^1^O_2_,·NO, HOCl; NO_3_^−^, NO_2_^−^; Fe^2+^, Cu^2+^, Cu^+^; Hcy, Cys, GSH	Peritonitis	RAW264.7 cells, mouse
[59]	**Xan42**	672	NR	<10 min	0.05–2	6.3	^1^O_2_, ClO^−^, H_2_O_2_, ·OH, NO, ·O_2_^−^, tBuOOH; SO_4_^2−^, Cl^−^, NO_2_^−^; Zn^2+^, Na^+^, K^+^, Mg^2+^, Ca^2+^, Cu^2+^; Glu, Cys, Glucose, BSA	Inflammatory	RAW264.7 and foam cells
[61]	**Dic1**	690	120	<20 min	0–180	4620	KO_2_, NO, ClO^−^, tBuOO·,·tBuOOH, H_2_O_2_, ·OH	Cell exogenous	HeLa cells
[62]	**Dic2**	670	NR	<20 min	0–10	53	HOCl, ^1^O_2_, ·O_2_^−^, tBuOOH, tBuOO·, H_2_O_2_, ·OH; NO_2_^−^, NO_3_^−^, F^−^, Cl^−^, Br^−^, CO_3_^2−^, H_2_PO_4_^−^, AcO^−^; Zn^2+^, Al^3+^, Fe^3+^, Cu^2+^, K^+^;·Hcy, Cys, GSH,	Cell endogenous	HepG2 cells
[63]	**Dic3**	678	NR	~25 min	10–200	78.7	·OH, NO, ClO^−^, ·O_2_^−^, tBuOO·, H_2_O_2_; S_2_O_3_^2−^; H_2_S,Hcy, Cys, GSH,	Cell endogenous	HepG2 cells
[64]	**Dic4**	685	NR	<10 min	0–20	96	·O_2_^−^, tBuOO·, H_2_O_2_, ·OH, NO, ClO^−^; NO_2_^−^, NO_3_^−^	Kainate (KA)-induced rat epilepsy	RAW264.7, HT22 cells, brain tissue, mouse
[65]	**Dic5**	660	30	<2 s	0–100	81	HNO, NO, ·OH, ·O_2_^−^, tBuOO·, H_2_O_2_, ClO^−^; F^−^, Cl^−^, I^−^, HCO_3_^−^, HSO_3_^−^, HS^−^, NO_2_^−^; Na^+^, K^+^, Fe^3+^, Cu^2+^; Tyr, Ala, Asp, Thr, Met, Ile, Phe, Hcy, Cys, GSH	Cell exogenous and endogenous	HeLa cells, zebrafish, mouse
[66]	**Dic6**	620	10	<4 min	1–6	27.5	NO, HNO, ClO^−^, ·OH, tBuOOH, ^1^O_2_, ·O_2_^−^; F^−^, Cl^−^, Br^−^, I^−^, AcO^−^, CO_3_^2−^, SO_4_^2−^, NO_2_^−^, NO_3_^−^; Hcy, Cys, GSH	Cell exogenous and endogenous	EC1 cells
[67]	**Dic7**	678	NR	<1 min	0–15	212	·OH, tBuOOH, ^1^O_2_, H_2_O_2_, ClO^−^; Cl^−^, Br^−^, I^−^, S^2−^, NO_3_^−^, NO_2_^−^, CO_3_^2−^, HSO_3_^−^, HCO_3_^−^, HSO_4_^−^; Fe^2+^, Cu^2+^, Fe^3+^, Na^+^, Ca^2+^, K^+^; Cys, GSH,	Parkinson’s disease	HeLa cells and zebrafish
[68]	**Dic8**	535	NR	<5 min	2–10	810	ClO^−^, ·OH, ^1^O_2_, ·O_2_^−^, H_2_O_2_; Cl^−^, AcO^−^, SO_4_^2−^, ClO_4_^−^, S^2−^, NO_3_^−^, NO_2_^−^,CO_3_^2−^, HSO_3_^−^; Mg^2+^, Zn^2+^, Na^+^, Ca^2+^, K^+^; Hcy, Cys, GSH	Idiopathic pulmonary fibrosis	BEAS cells, mouse
[69]	**Dic9**	657	NR	<100 s	0–20	5300	ClO^−^, ·OH, ·O_2_^−^, H_2_O_2_; SO_3_^2−^, AcO^−^, SO_4_^2−^, Cl^−^, NO_2_^−^; Al^3+^, Fe^2+^, Cu^2+^, Na^+^, Ca^2+^, K^+^	Cell exogenous and endogenous	HeLa, Raw264.7 and HepG2 cells, zebrafish
[70]	**Dic10**	667	50	<5 min	0–270	NR	tBuOO·, ·OH, ·O_2_^−^, ^1^O_2_, ClO^−^, H_2_O_2_	Cell exogenous	HeLa cells
[71]	**Dic11**	660	NR	<3 s	0–15	5	·OH, tBuOOH, ClO^−^, H_2_O_2_, tBuOO·, NO, ·O_2_^−^, ^1^O_2_; NO_3_^−^, Cl^−^, NO_2_^−^, SO_4_^2−^; Fe^2+^, Cu^2+^, Fe^3+^, Na^+^, Ca^2+^, K^+^, Mg^2+^, Zn^2+^, Cys, GSH, Hcy	Cell exogenous and endogenous	RAW264.7 cells and zebrafish
[72]	**Dic12**	650	30	<5 s	0–20	53	tBuOOH, NO, ·OH, ·O_2_^−^, tBuOO·, ^1^O_2_, ClO^−^, H_2_O_2_; HSO_3_^−^, SO_3_^2−^, NO_2_^−^; Cys	Inflammation	HepG2 cells and mouse
[73]	**Dic13**	560	NR	<25 s	0–10	130	NO, ClO^−^, ^1^O_2_, ·O_2_^−^, H_2_O_2_, ·OH; Cl^−^, S^2−^, NO_3_^−^, NO_2_^−^, CO_3_^2−^, AcO^−^, SO_3_^2−^; Na^+^, Ca^2+^, K^+^, Mg^2+^, Al^3+^, Fe^2+^, Cu^2+^; Cys, GSH, Hcy	Acute liver injury	LX-2 cells and mouse
[74]	**Dic14**	670	NR	<1 s	0–20	4.59	·O_2_^−^, tBuOO·, ·OH, ^1^O_2_, ClO^−^, H_2_O_2_, NO, BrO^−^; HS^−^, SO_4_^2−^, HSO_3_^−^, SO_3_^2−^, S^2−^, CO_3_^2−^, NO_3_^−^, NO_2_^−^; Zn^2+^, Na^+^, Ca^2+^, K^+^, Mg^2+^; Hcy, Cys, GSH	Parkinson’s disease	PC12 and SH-SY5Y cells, tissues, drosophila brains, mouse
[76]	**Cou1**	630	8	<10	0–50	34	tBuOOH, ·O_2_^−^, ·OH, ^1^O_2_, ClO^−^, H_2_O_2_, NO; NO_2_^−^, NO_3_^−^, CO_3_^2−^, SO_4_^2−^, SO_3_^2−^, PO_4_^3−^; Fe^3+^, Zn^2+^, Fe^2+^, Cu^2+^, Na^+^, Ca^2+^, K^+^; H_2_S, Hcy, Cys, GSH, vitamin C	Anthracycline-induced cardiotoxicity	H9c2 cardiomyocytes and mouse
[77]	**Cou2**	510	25	<60	0–40	21.4	ClO^−^, ·NO, ·O_2_^−^, ^1^O_2_, H_2_O_2_; F^−^, ClO_4_^−^, Cr_2_O_7_^2−^, S_2_O_3_^2−^, I^−^, S^2−^, CO_3_^2−^, NO_3_^−^; Ca^2+^, K^+^, Cd^2+^, Mn^2+^, Cu^2+^, Ni^2+^, Ba^2+^, Al^3+^, Mg^2+^, Hg^2+^, Cr^3+^, Zn^2+^, Ag^+^	γ-carrageenan-induced inflammation	RAW264.7 cells and mouse
[78]	**Cou3**	628	8	<5 s	0.064–0.64	3.7	ClO^−^, H_2_O_2_, NO, ·OH, ·O_2_^−^, HNO; NO_2_^−^, SCN^−^, HSO_3_^−^, HS^−^; K^+^, Mg^2+^; Cys, GSH, FA	Cell exogenous and endogenous	SMMC-7721 and RAW264.7 cells
[79]	**Cou4**	520	111	<5 min	7–16	210	HClO, tBuOO·, tBuOOH, ·OH, ^1^O_2_, NO, H_2_O_2_; NO_2_^−^, AcO^−^, SO_4_^2−^, CO_3_^2−^, S_2_O_3_^2−^, S^2−^, SCN^−^; Zn^2+^, Ca^2+^, Mg^2+^, Fe^2+^, Cu^2+^, Na^+^, K^+^; Hydrazine hydrate, Cys, Hcy, GSH	Cell exogenous and endogenous	MCF cells and HepG2 cells
[80]	**Cou5**	480	76	<1 min	0–10	35	·O_2_^−^, tBuOO·, ·OH, ^1^O_2_, NO, HClO, tBuOOH, H_2_O_2_; NO_2_^−^, NO_3_^−^; Hcy, Cys, GSH	Cell exogenous and endogenous	RAW264.7 and H1299 cells
[81]	**Cou6**	538	153	<3 min	0–18	16	ClO^−^, ·OH, ^1^O_2_, tBuOOH, ·O_2_^−^, H_2_O_2_, HNO; NO_3_^−^, NO_2_^−^; Ca^2+^, Mg^2+^, Fe^2+^, Cu^2+^; H_2_S_2_	Cell endogenous	HepG2 cells
[82]	**Cou7**	650	NR	<5 s	0–15	53.8	^1^O_2_, ·O_2_^−^, HNO, NO, ClO^−^, H_2_O_2_, ·OH; SO_3_^2−^, N_3_^−^, HSO_4_^−^, NO_2_^−^, Br^−^, CN^−^, F^−^, Cl^−^; Cys, Hcy, GSH	Cell exogenous and endogenous	Hela cells
[83]	**Cou8**	450	NR	<4 min	0–10	29.8	ClO^−^, ·OH, ·O_2_^−^, NO, H_2_O_2_, tBuOOH, tBuOO·; NO_3_^−^, NO_2_^−^; IAA, Trp, Glu, BSA, HSA	High-fat diet-induced obese	RAW264.7 and EAhy926 cells, zebrafish and in live tissues
[84]	**Cou9**	500	1200	<2 s	0–2	70.8	NO, ClO^−^, ·OH, tBuOOH; Fe^3+^, Fe^2+^, Ca^2+^, Cu^2+^, Al^3+^, Hg^2+^, Pb^2+^, Mg^2+^, Zn^2+^; HNO_3_, GSH, Cys, Hcy	Cell exogenous and endogenous	HepG2 and HL772 cells
[85]	**NCou1**	540	NR	<30 min	3–10	2500	·O_2_^−^, ·NO, H_2_O_2_, tBuOO·, ClO^−^, ·OH, tBuOOH	Cell exogenous and endogenous	J774A.1cells
[86]	**NCou2**	530	NR	<20 min	0–10	15	·O_2_^−^, H_2_O_2_, tBuOO·,·OH, tBuOOH, ClO^−^; Fe^3+^, Ca^2+^, Cu^2+^, Zn^2+^; Cys, Glu	Drug-damaged liver	HepG2 cells and mouse
[87]	**NCou3**	525	24	<50 min	10–35	30	^1^O_2_, HNO, ·OH, ·O_2_^−^, tBuOOH, ClO^−^, H_2_O_2_; Zn^2+^, S^2−^, NO_2_^−^, NO_3_^−^; Ca^2+^, Mg^2+^, Na^+^, K^+^, Fe^3+^; GSH, Cys, Hcy	Cell exogenous and endogenous	HeLa cells and mouse
[88]	**NCou4**	522	155	50 s	0–5	0.83	·OH, NO, ·O_2_^−^, H_2_O_2_, ClO^−^, ^1^O_2_; NO_2_^−^, SO_4_^2−^, H_2_PO_4_^−^, I^−^, HCO_3_^−^, Br^−^, F^−^; Fe^2+^, Cu^2+^, Zn^2+^, Ca^2+^, Mg^2+^, Na^+^, K^+^	Cell exogenous and endogenous	RAW264.7 cells
[90]	**Nap1**	550	NR	<70 s	0–1000	130	ClO^−^; SCN^−^, F^−^, Cl^−^, NO_3_^−^, I^−^, HPO_4_^2−^, CO_3_^2−^, HSO_4_^−^, SO_4_^2−^; K^+^, Li^+^, Ba^2+^, Al^3+^, Fe^2+^, Pb^2+^, Cu^2+^, Ca^2+^, Mg^2+^; Asn, Arg, Leu, Trp	Acute liver injury	LX-2 cells, mouse
[91]	**Nap2**	558	NR	<6 s	2–15	69	HNO, ·OH, NO, ·O_2_^−^, H_2_O_2_, ClO^−^; S^2−^, SO_3_^2−^, I^−^; Zn^2+^, Ca^2+^, Fe^2+^; CO, vitamin C, Cys, Hcy, GSH	Cell exogenous and endogenous	Hela and HepG2 cells, mouse
[92]	**Nap3**	550	NR	<100 s	0–20	69	HClO, tBuOO·,^1^O_2_, ·OH, ·O_2_^−^, tBuOOH, NO, H_2_O_2_; Cl^−^, SO_4_^2−^, NO_3_^−^, NO_2_^−^, S^2−^; Fe^3+^, Cu^2+^, Fe^2+^, Zn^2+^, Mg^2+^, Na^+^, K^+^, Ca^2+^; Cys, Hcy, GSH	Cell exogenous and endogenous	RAW264.7 cells and zebrafish
[93]	**Nap4**	548	NR	<12 min	10–80	49.7	^1^O_2_, ClO^−^, ·OH, ·O_2_^−^, tBuOOH, NO, H_2_O_2_; Cl^−^, HSO_3_^−^, SO_4_^2−^, S_2_O_3_^2−^, NO_2_^−^, NO_3_^−^; Fe^3+^, Cu^2+^, Fe^2+^, Mg^2+^, Na^+^, K^+^, Ca^2+^; Cys, Hcy, GSH	Cell exogenous and endogenous	HepG2 cells and *C. elegans*
[94]	**Nap5**	553	NR	<200 s	0–44	48	ClO^−^, ·OH, ·O_2_^−^, NO, H_2_O_2_; SO_3_^2−^; Zn^2+^, Mg^2+^, Fe^3+^, Cu^2+^, Fe^2+^, Na^+^, K^+^; H_2_S, H_2_S_n_, Cys, Hcy, GSH, BSA, DNA, erastin	Ferroptosis	HepG2 cells and zebrafish
[95]	**Nap6**	550	4	<1 min	0–10	184	ClO^−^, ·OH, NO·, H_2_O_2_, tBuOO·, tBuOOH	Cell exogenous and endogenous	SH-SY5Y cells and mouse
[96]	**Nap7**	565	NR	<120 s	0–18	NR	^1^O_2_, ·OH, ·O_2_^−^, ClO^−^, tBuOOH, H_2_O_2_, NO; Cys, Hcy, GSH, H_2_S, Aβ_42_ peptide, BSA, DNA	Alzheimer’s disease	PC12 cells and mouse
[97]	**Nap8**	545	15	<5	0–20	320	ClO^−^, ^1^O_2_, ·OH, ·O_2_^−^, tBuOOH, tBuOO·, H_2_O_2_; HS^−^, ClO_3_^−^, HCO_3_^−^, SO_4_^2−^, ClO^−^, SO_3_^2−^, CO_3_^2−^, NO_3_^−^, NO_2_^−^, Br^−^, H_2_PO_4_^−^, I^−^, F^−^, Cl^−^; Cys, Hcy, GSH	Cell exogenous and endogenous	RAW264.7 cells and zebrafish
[103]	**Cy1**	560	324	<30 s	0–0.7	0.65	ClO^−^, ^1^O_2_, ·OH, ·O_2_^−^, tBuOOH, H_2_O_2_; HSO_4_^−^, SO_3_^2−^; Cys, Hcy, GSH	Cell exogenous and endogenous	RAW264.7 cells,
[104]	**Cy2**	610	46	<20 min	0–30	280	HClO, ^1^O_2_, ·OH, ·O_2_^−^, NO, H_2_O_2_; NO_2_^−^, HS^−^; Fe^3+^, Fe^2+^, Mg^2+^, Na^+^, K^+^, Ca^2+^, Zn^2+^; Cys, Hcy, GSH	Cell endogenous	HeLa cells
[105]	**Cy3**	NR	NR	NR	0–3.3	26	ClO^−^, ·OH, ·O_2_^−^, H_2_O_2_; NO_3_^−^, NO_2_^−^	Cell exogenous and endogenous	RAW264.7 cells
[106]	**Cy4**	950	NR	<3 min	0–11	55.9	^1^O_2_, NO, ClO^−^, ·OH, ·O_2_^−^, H_2_O_2_; HS^−^, NO_2_^−^; Na^+^; Cys	APAP-induced hepatotoxicity	Mouse
[107]	**Cy5**	719	41	<5 min	0–35	25.4	NO, ClO^−^, H_2_O_2_; NO_3_^−^, NO_2_^−^; Fe^3+^, Fe^2+^, Mg^2+^, Ca^2+^, Zn^2+^ Cu^2+^, Cd^2+^, Ag^+^; Cys, Hcy, GSH	Stroke-induced oxidative stress	PC12 cells and BV-2 cells, and mouse
[108]	**Cy6**	630	NR	<15 min	1–100	9.2	^1^O_2_, ·O_2_^−^, NO, ·OH, ClO^−^, H_2_O_2_; HS^−^, NO_2_^−^; Na^+^; S-nitrosoglutathione, methyl linoleate hydroperoxide	Hypoxic stress	LO2 cells, zebrafish, mice
[110]	**Cy7**	460	1728	<60 s	0.1–15	33	tBuOO·, ·OH, ^1^O_2_, ClO^−^, H_2_O_2_, NO; SO_4_^2−^, HSO_3_^−^, NO_3_^−^, NO_2_^−^; H_2_S, Hcy, Cys, GSH	Cell exogenous and endogenous	RAW264.7 cells
[111]	**Cy8**	484	448	<10 min	0.5–15	77	tBuOOH, HClO, ·O_2_^−^, H_2_O_2_; N_3_^−^, NO_3_^−^, NO_2_^−^, HSO_3_^−^, SO_3_^2−^; H_2_S, Hcy, Cys, GSH	Cell exogenous and endogenous	HepG2 cells
[112]	**Cy9**	456	NR	<3 min	0–30	326	·OH, ^1^O_2_, ClO^−^, H_2_O_2_; F^−^, Cl^−^, Br^−^, I^−^, AcO^−^, ClO_4_^−^, HPO_4_^2−^, SO_4_^2−^, S_2_O_3_^2−^, NO_2_^−^, NO_3_^−^, HCO_3_^−^, CO_3_^2−^, H_2_PO_4_^−^; Na^+^, K^+^; Hcy, Cys, GSH	Cyclophosphamide-induced oxidative stress	HeLa cells
[113]	**Cy10**	560	NR	<15 min	0–100	210	NR	Cell exogenous and endogenous	HepG2 cells
[114]	**Cy11**	530	NR	<4 min	0–12	84	·OH, ·O_2_^−^, tBuOOH, tBuOO·, H_2_O_2_, ClO^−^; S^2−^, HS^−^, S_2_O_3_^2−^, HSO_3_^−^, NO_3_^−^, NO_2_^−^; Na^+^, K^+^, Zn^2+^, Fe^3+^, Fe^2+^, Mg^2+^, Ca^2+^; Cys, Hcy, GSH	Cell exogenous and endogenous	HeLa cells
[115]	**Cy12**	535	NR	<2 min	5–50	85	NO, ·OH, ^1^O_2_, tBuOOH, HClO, ·O_2_^−^, H_2_O_2_; Cys, GSH	Idiopathic pulmonary fibrosis	A549 and RAW264.7 cells, mouse
[116]	**Cy13**	444	NR	<20 s	0–20	40	NO, ·OH, ·O_2_^−^, H_2_O_2_, ClO^−^, ^1^O_2_; S^2−^, NO_3_^−^, NO_2_^−^; Cys, Hcy, GSH	Cell exogenous and endogenous	HepG2 cells
[117]	**Cy14**	635	NR	<250 s	0–18	78	NO, ·OH, ·O_2_^−^, H_2_O_2_, ClO^−^, ^1^O_2_; S_2_O_3_^2−^, NO_2_^−^; Na^+^, Zn^2+^, Fe^3+^, Ca^2+^; Cys, GSH, citric acid	Tunicamycin -induced endoplasmic reticulum stress	HeLa cells and zebrafish
[118]	**Cy15**	493	25	<4 min	0–20	150	ClO^−^, ^1^O_2_, ·OH, ·O_2_^−^, tBuOOH, H_2_O_2_, NO; NO_3_^−^, NO_2_^−^	Cell endogenous	RAW264.7 cells
[119]	**Cy16**	515	474	NR	0–20	49.7	NO, ·OH, ·O_2_^−^, H_2_O_2_, ClO^−^, tBuOOH, tBuOO·; Cys, Hcy, GSH	Cell exogenous and endogenous	WI38 VA13 and RAW264.7 cells
[120]	**Cy17**	505	22	<2 s	0–40	67	NO, ·OH, ·O_2_^−^, H_2_O_2_, ClO^−^, tBuOO·, HNO; NO_2_^−^, HSO_3_^−^, SO_3_^2−^, Cl^−^, S_2_O_3_^2−^, HS^−^; Na^+^, Fe^2+^, Mg^2+^, Ca^2+^, Zn^2+^ Cu^2+^; Cys, Hcy, GSH	Nonalcoholic fatty liver	Hela and RAW264.7 cells, mouse
[121]	**Cy18**	500	11	<3 min	0–10	16	ClO^−^, ^1^O_2_, ·OH, ·O_2_^−^, tBuOOH, H_2_O_2_, NO; S^2−^, NO_3_^−^, NO_2_^−^, AcO^−^, HSO_4_^−^, Cl^−^, SO_4_^2−^, HSO_3_^−^; Na^+^, K^+^, Zn^2+^, Fe^3+^, Fe^2+^, Mg^2+^, Ca^2+^, Cu^2+^; Cys, Hcy, GSH	Hepatotoxicity induced by acetaminophen	HepG2 cells and zebrafish
[122]	**Cy19**	477	125	<10 s	0–2	13	tBuOO·, HNO, ·OH, NO, KO_2_, H_2_O_2_; HSO_4_^−^, F^−^, Cl^−^, Br^−^, I^−^, AcO^−^, S_2_O_3_^2−^, HCO_3_^−^, CO_3_^2−^, C_2_O_4_^2−^, HS^−^, HSO_3_^−^, S_2_O_7_^−^;Na^+^, K^+^, Ca^2+^; Ser, Val, Lys, Trp, Gly, Ala, GSH, Hcy, Cys	Golgi oxidative stress and drug-induced liver injury	Hela cells and mouse
[123]	**Cy20**	484	52	<5 min	0–3	41.88	tBuOOH, HClO, H_2_O_2_, ^1^O_2_, NO^−^; HSO_3_^−^, HPO_4_^2−^, SO_4_^2−^, S_2_O_3_^2−^, NO_3_^−^; Fe^2+^, Na^+^; Cys, Hcy, GSH	Cell endogenous	HepG2 cells
[124]	**Cy21**	680	NR	<90 min	0–40	56	ClO^−^, ^1^O_2_, ·OH, ·O_2_^−^, tBuOOH, H_2_O_2_; NO_2_^−^, CN^−^, HSO_3_^−^, NO_3_^−^	Cell exogenous and endogenous	RAW264.7 cells, zebrafish, live mouse tissues
[125]	**Cy22**	505	120	<40 min	0–80	13	ClO^−^, ^1^O_2_, ·OH, ·O_2_^−^, tBuOO·, H_2_O_2_	Rheumatoid arthritis	RAW264.7 cells and mouse
[126]	**Cy23**	576	32	<120 s	0–16	60.5	ClO^−^, NO·, ·O_2_^−^, ·OH, H_2_O_2_, tBuOO·, tBuOOH; K^+^, Na^+^, Ca^2+^, Mg^2+^, Pb^2+^, Mn^2+^, Zn^2+^, Cu^2+^, Fe^2+^, Fe^3+^, Mn^2+^, Cd^2+^, Li^+^	Inflammatory	RAW264.7 cells and zebrafish
[127]	**Cy24**	605	NR	<10 min	8–48	250	ClO^−^, ^1^O_2_, ·OH, H_2_O_2_; NO_3_^−^, NO_2_^−^, ClO_4_^−^, AcO^−^, SO_3_^2−^, HCO_3_^−^, CO_3_^2−^, HSO_3_^−^, S^2−^; Na^+^, K^+^, Zn^2+^, Fe^3+^, Mg^2+^, Ca^2+^, Cu^2+^; Cys, Hcy, GSH	Cell exogenous and endogenous	RAW264.7 cells and zebrafish
[128]	**Cy25**	557	NR	<10 min	0–15	32	ClO^−^, OH, ·O_2_^−^, H_2_O_2_; Na^+^, K^+^, Al^3+^, Zn^2+^, Fe^3+^, Ca^2+^, Cu^2+^; SO_4_^2−^, Cl^−^, NO_2_^−^, CO_3_^2−^	Drug-induced liver injury	RAW264.7 cells and zebrafish
[129]	**Cy26**	569	NR	<1 min	0–10	16	tBuOO, ·tBuOOH, ClO^−^, OH, ^1^O_2_, H_2_O_2_; NO_3_^−^, NO_2_^−^, HSO_4_^−^, Cl^−^, Br^−^, I^−^, S^2−^, HCO_3_^−^, CO_3_^2−^, HSO_3_^−^; Na^+^, K^+^, Fe^2+^, Fe^3+^, Ca^2+^, Cu^2+^; GSH, Cys, Ascorbic acid	Cell exogenous and endogenous	Hela cells
[130]	**Cy27**	712	59	<2 min	0–10	53	ClO^−^, OH, ·O_2_^−^, H_2_O_2_, ^1^O_2_	Tumor	RAW264.7 cells and mouse

Note: Cys = cysteine, Met = methionine, GSH = glutathione, Hcy = homocysteine, Gly = glycine, Leu = leucine, Lys = lysine, Val = valine, Glu = glutamine, Tyr = tyrosine, Asp = aspartic acid, Phe = phenylalanine, Asn = asparagine, Ser = serine, Ile = isoleucine, Arg = arginine, His = histidine, Trp = tryptophan, Thr = threonine, Pro = proline, NOC-9 = mahma-nonoate, FA = folic acid, IAA = indole-3-acetic acid, BSA = bovine albumin, HAS = human serum albumin, NR = not reported.

## 3. Summary and Outlook

In conclusion, we systemically reviewed over 100 peroxynitrite-responsive fluorescent probes based on their fluorophore core. The response pathways, in vivo peroxynitrite response data, bio-system peroxynitrite produce mode, and fluorescent bioimaging objects of the probes were summarized in detail. Based on the overall experimental results, specific and sensitive detection of peroxynitrite could be achieved for most of the reported probes. Many of the probes have been applied to reveal the important role of peroxynitrite in a great diversity of disease processes.

Although the number of articles concerning peroxynitrite responsive fluorescent probes has appeared to have had an explosive increase in the last 6 years and remarkable progress has been achieved, the design and application of a new class of fluorophore core, new responsive moiety, new application mode, and probes with higher potential in clinical translation are still challenging and greatly required.

## Data Availability

Not applicable.
